# The Impact of Physical Disturbance and Increased Sand Burial on Clonal Growth and Spatial Colonization of *Sporobolus* *virginicus* in a Coastal Dune System

**DOI:** 10.1371/journal.pone.0072598

**Published:** 2013-08-19

**Authors:** Elena Balestri, Claudio Lardicci

**Affiliations:** Department of Biology, University of Pisa, Pisa, Italy; Beijing Forestry University, China

## Abstract

Dune plants are subjected to disturbance and environmental stresses, but little is known about the possible combined effects of such factors on growth and spatial colonization. We investigated how clones of *Sporobolus* *virginicus* , a widespread dune species, responded to the independent and interactive effects of breakage of rhizomes, breakage position and burial regime. Horizontal rhizomes were severed at three different internode positions relative to the apex to span the range of damage by disturbance naturally observed or left intact, and apical portions exposed to two burial scenarios (ambient vs. increased frequency) for three months in the field. The performance of both parts of severed rhizomes, the apical portion and the remaining basal portion connected to clone containing four consecutive ramets, was compared with that of equivalent parts in intact rhizomes. Apical portions severed proximal to the third internode did not survive and their removal did not enhance branching on their respective basal portions. Severing the sixth or twelfth internode did not affect survival and rhizome extension of apical portions, but suppressed ramet production and reduced total biomass and specific shoot length. Their removal enhanced branching and ramet production on basal portions and changed the original rhizome growth trajectory. However, the gain in number of ramets in basal portions never compensated for the reduction in ramet number in apical portions. Recurrent burial increased biomass allocation to root tissues. Burial also stimulated rhizome extension only in intact rhizomes, indicating that disturbance interacts with, and counteracts, the positive burial effect. These results suggest that disturbance and recurrent burial in combination reduces the regeneration success and spread capacity of *S* *. virginucus* . Since global change leads to increasingly severe or frequent storms, the impact of disturbance and burial on clones could be greater in future and possibly prevent colonization of mobile dunes by the species.

## Introduction

Disturbance may result in a partial or complete removal of above-ground biomass, and is considered as an important determinant of plant abundance and distribution [1,2]. In coastal sand dunes, plants are often simultaneously exposed to mechanical disturbances (e.g. winds, waves and grazing) and environmental stresses (e.g. sand burial and salt spray) [3–6]. The ability to tolerate, or respond to a disturbance event by rapid regrowth may be therefore crucial for the persistence of species in such habitats.

Rhizomatous clonal plants are a dominant component of communities in coastal dunes and play a fundamental role in stabilizing sand. Physiological integration, the ability to share resources such as carbohydrates, water and nutrients between ramets interconnected by rhizome or stolons, can improve the ability of these plants to cope with natural disturbances and local environmental stresses such as nutrient deficiency and sand burial [7–11]. Yet, little is known about the possible interactive effects of such factors on clonal growth and pattern of spatial colonization [12–14]. Rhizomatous plants mainly rely on the growth and branching of horizontal rhizomes to colonize bare areas [15–17]. Such rhizomes are vulnerable to disturbance, especially autumn-winter storms, which can mechanically break rhizome at different distances relative to the apex, generating isolated ramets or groups of interconnected ramets at various ontogenetic stages of maturation.

To date, available data on the severity of physical disturbance in terms of number of ramets separated from the parental axis are scarce [18–20], and its possible impact on the growth of a damaged clone remained to be elucidated for most species. Understanding fully the impact of this type of disturbance is quite complex because both the parts of a damaged rhizome, the apical portion disconnected from the parent clone and remaining basal portion connected to the parent clone, have the potential to survive and generate new ramets after disturbance. However, once separated for the parent clone, apical rhizome portions could suffer from decreased growth compared to equivalent parts in intact rhizomes because of the lack of internal support through physiological integration [9,12–14]. The extent of such impact may vary among species according to the degrees of physiological integration between ramets, and may depend in a given species on the size of the removed portions at the time of disturbance [12–14], which in turn is related to the location of the break within a rhizome. Indeed, in clonal species both the production of new ramets (younger ramets) by apical meristem of horizontal rhizomes and their growth are physiologically dependent upon assimilates translocated from older connected ramets [21–23]. This dependence declines as the ramets mature and acquire physiological autonomy, although the stage of maturation at which a ramet becomes potentially independent of the rest of a clone is unknown for most species. However, the intensity and duration of physiological integration may be dependent of the intensity and duration of the stress experienced for the clone. For example, a ramet growing in stressful (and perhaps lethal) conditions could maintain active physiological integration with the parts of the clone under more favourable conditions indefinitely [21,23]. The effect of disturbance is therefore expected to be more consistent for smaller apical portions, i.e., those resulting from the breakage of rhizomes in close proximity of the apical meristem, but local environmental conditions may interact with disturbance to further affect rhizome growth.

Sand burial is a major abiotic stress experienced by plants in coastal mobile dunes. Most dune plants can withstand gradual burial by wind by elongating vertical structures, but many species cannot recover from recurrent sudden burial episodes even of moderate intensity (> 50% of plant height) [3,6,24–26]. Previous studies have showed that small fragments consisting of a single node had a lower probability of emergence from deep burial than those with multi-node fragments probably because of an insufficient amount of resources stored in their rhizome [19,20]. Predictions indicate that global change will increase the frequency and/or intensity of winds in the coming decades [27–30], leading to greater sediment instability. Consequently, only larger apical portions are expected to survive and eventually initiate the formation of new patches after disturbance under increasing burial conditions. On the other hand, the removal of an apical portion from the horizontal rhizome could stimulate the production of lateral branches by buds closest to the point of breakage situated on the basal portion connected to clone to compensate for the loss of tissue with no overall effect on spread of the damaged clone. Enhanced branching after artificial removal of the rhizome or stolon apex caused by the release from apical dominance has been documented for some clonal species, and is regarded as a compensatory mechanism to ensure a continuation of growth after natural disturbances [31–34]. However, if the tissue removed by disturbance is excessive or the portion is large enough to contain all nodes that are influenced by apical dominance, this mechanism could be insufficient to fully compensate for the loss and thus could reduce the spatial colonization of the damaged clone. Therefore, understanding of interaction of disturbance, burial regime and regeneration of both segments of a severed rhizome is particularly important to formulate predictions about the consequences of climate-induced alterations for dune plant populations and to develop effective conservation strategies.

In the present study, we hypothesized that disturbance in combination with burial substantially affected the growth of horizontal rhizomes (interactive effect). We tested this hypothesis in a field factorial experiment with *Sporobolus* *virginicus* Schreber Kunth. This perennial pioneer species was selected because of its worldwide distribution and production of horizontal rhizomes up to several metres in length that give rise to extensively spreading clones on mobile dunes. The performance (survival, growth and biomass partitioning) of apical portions severed at three different positions (i.e., number of internodes from the apex) within horizontal rhizomes to simulate different degrees of disturbance was compared to that of equivalent portions in intact rhizomes subjected to two burial regimes (ambient vs. increased frequency and intensity) for three months. We predicted that rhizome damage by disturbance would reduce the growth of isolated apical portions, especially smaller portions, and decrease the probability of successful establishment of new patches under increased burial conditions because it deprived ramets from internal support through physiological integration. In addition, in a parallel experiment we examined the growth and branching capacity of nodes on the basal rhizome portion remained connected to parent clones after severing apical portions at the three different positions. We expected that any reduction in growth due to the severing of the apical portions would be matched by increased growth of the corresponding basal portions because of apical dominance release, and this would affect the architecture and spatial colonization potential of damaged clones. To our knowledge, the effects of different degrees of disturbance on the subsequent growth of both parts of severed horizontal rhizomes of dune plants have not previously studied in the field.

## Materials and Methods

### Ethics approval

*Sporobolus* *virginicus* is not a protected or endangered species in Italy. The study system belongs to Rosignano Marittimo Municipality. All necessary permits were obtained from this Municipality for the described study, which complied with all relevant regulations.

### Plant species and site description

*Sporobolus* *virginicus* (Poaceae) is a common pioneer grass [35]. The dune ecotype (coastal dropseed) produces horizontal rhizomes with long internodes, branched or solitary erect culms that stabilize mobile dunes. This species is capable of forming adventitious roots from each node sited along the horizontal rhizome. It can reproduce sexually but seedlings have been rarely observed on mobile dunes [36–38], and thus clonal growth seems to play a major role in maintaining populations and colonizing dune habitats. Available data on the growth pattern of the species are scarce [39–41], and virtually nothing is known on the degree of physiological integration and apical dominance.

The study was conducted in mobile dunes near Rosignano Solvay, Italy (43°22'43.10″N, 10°26'15.77″E). These dunes are dominated by *Ammophila* *arenaria* (L.) Link (European beach grass) and *Elymus* *farctus* (Viv.) Runemark ex Melderis. *S* *. virginicus* is abundant on the first dune ridges and upper beach where it forms monospecific stands. The climate is typically Mediterranean: the mean daily temperature of the coldest month (January) is 5 °C, while that of the warmest month (July) is 25°C. The frequency of wind storms has considerably increased during the past decades [42]. Data from local meteorological stations (available from http://www.ilmeteo.it) indicate that the present day frequency of strong winds was 1-2 events month^-1^. After the most recent storm at the dune system and preceding our study (9 January 2010, max wind speed 23 m s^-1^), naturally damaged horizontal rhizome fragments of *S* *. virginicus* (*n* = 30) with apex were randomly sampled along a stretch (500 meters long and 10 metres wide) of the mobile dune system: The length and number of internodes in each fragment were recorded to determine the location of breaks relative to the apex and the extent of damage (as number of ramets in detached fragments) that naturally occurred most frequently. This information enabled us to determine the levels of rhizome severing (i.e. internode position along the rhizome relative to the apex) to apply in the field experiment to simulate the effects of natural physical disturbance.

### Experimental design

The study was conducted from September to November 2010 during the growing season of the species. In September 2010, three plots (blocks) with similar distance from the shoreline and elevation (1.5-2 m from the 0 m water level) were established in an area of approximately 1.5 ha. In each block, 18 patches were randomly selected. In each patch, one horizontal rhizome of *S* *. virginicus* with an intact apical nonbranched region containing at least 16 ramets (the term ramet was used here to refer as a node, the attached shoot and roots if present, and the internode immediately distal to the node) was selected, so there were 18 rhizomes in each block. These rhizomes were divided into two groups of 12 and six rhizomes respectively and the groups were assigned to one of the two simultaneous experiments.

**Figure 1:**
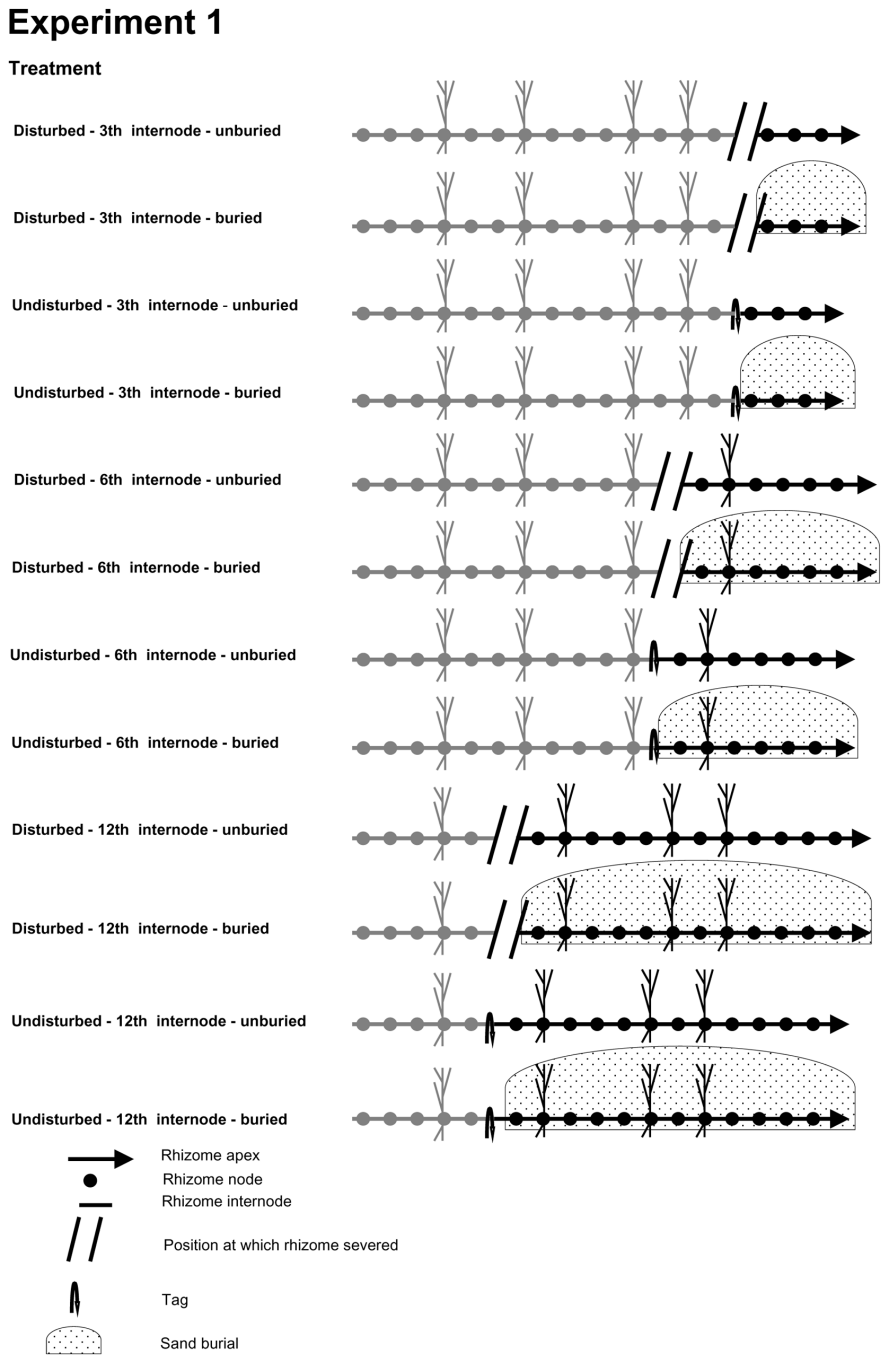
Diagram of the experiment 1. Diagrammatic presentation of *Sporobolus* *virginicus* horizontal apical rhizomes segments consisting in rhizome interconnections and nodes, at the start of the experiment. In the disturbed treatments, the position (third, sixth or twelfth internode from the apical meristem) at which the rhizome was artificially disconnected from the parent clone is indicated with parallel lines. In the control (undisturbed), the point at which the rhizome was tagged is indicated with a curved arrow. The apical portion considered in this experiment is showed in black whereas the rest of the rhizome portion connected to parent clone is showed in grey. In the burial treatments, the apical rhizome portion covered by sand is indicated with a shadowed area.

In one experiment (Figure 1), we evaluated the effects of the breakage of rhizome, breakage position (i.e. position of the severed internode within the rhizome relative to the apex) and burial regime (ambient vs. increased frequency and intensity) on the performance of apical rhizome portions. To this end, six rhizomes in each block were randomly selected for breakage (hereafter referred to as disturbed rhizomes) and other six rhizomes were left intact (hereafter referred to as undisturbed rhizomes). In disturbed rhizomes, the connections were severed with scissors at three different positions relative to the apex (third, sixth or twelfth internode) chosen to span the range of damage by disturbance naturally observed, and apical portions were uprooted and replanted immediately to simulate storm events. Apical portions, therefore, included the rhizome apex and either two consecutive ramets (hereafter referred to as short apical portions), five consecutive ramets (hereafter referred to as intermediate apical portions) or 11 consecutive ramets (hereafter referred to as long apical portions). Undisturbed rhizomes were left intact and tagged with a cotton thread placed around the internodes situated at the same distances from the apex as severed ones. Apical portions of disturbed and undisturbed rhizomes were then randomly assigned to one of two burial treatments, increased burial frequency and intensity and ambient conditions. In the burial treatment, apical rhizomes were completely buried with a 6 cm deep layer of sand. No sand was added to rhizome left at ambient conditions. The sand used for the burial treatment was collected closely to the treated plants; it was sieved to remove propagules and extraneous material prior to the use. Sand height was weekly monitored, and apical rhizomes were reburied with sand to the experimental originally attributed height. The imposed burial height was equalled to the maximum sand accretion level previously recorded at the study site while the burial frequency was about two folds than the present day [41]. Before burial treatment application, the length of the rhizome, the number of vertical shoots and the height of the longest shoot were recorded. The number of roots of each apical portion was also determined by visual inspection after gently removing sand from the rhizome. Apical portions that included two interconnected ramets did not have developed shoots and roots. Intermediate apical portions containing five interconnected ramets had originally on average 1.1 (± 0.4) shoots and 0.7 (± 0.3) roots, and the length of the longest shoot was 1.9 (± 0.7) cm. Long apical portions containing eleven interconnected ramets had on average 1.5 (± 0.6) shoots and 0.9 (± 0.3) roots, and the length of the longest shoot was 1.7 (± 0.6) cm. Preliminary analyses demonstrated that apical portions assigned to the different treatment combinations were homogenous in size and similar in morphological characters at the start of the experiment (Pseudo-*F*_3,8_ = 1.74, *P* = 0.11 for intermediate portions and Pseudo *F*_3,8_ = 1.63*, P* = 0.12 for long portions).

**Figure 2:**
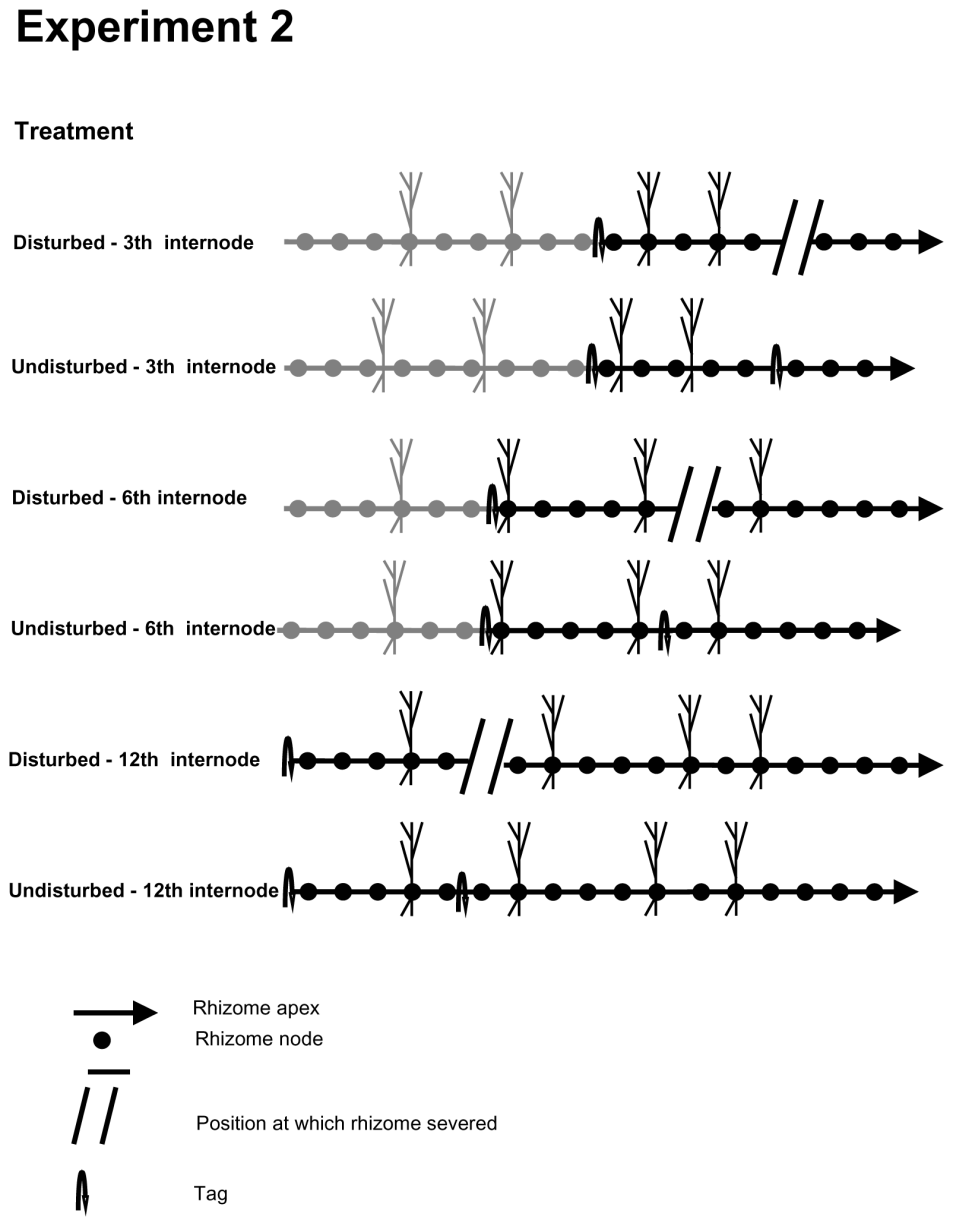
Diagram of experiment 2. Diagrammatic presentation of *Sporobolus* *virginicus* horizontal apical rhizomes segments consisting in rhizome interconnections and nodes, at the start of the experiment. In the disturbed treatments, the position (third, sixth or twelfth internode from the apical meristem) at which the rhizome was artificially disconnected from the parent clone is indicated with parallel lines. In the control (undisturbed), the point at which the rhizome was tagged is indicated with a curved arrow. In disturbed treatments, the basal portion remained attached to the parent clone which was considered in this experiment is comprised between parallel lines and curved arrow. In the control (undisturbed), the basal portion remained attached to the parent clone is comprised between two consecutive curved arrows.

In the other experiment (Figure 2), we examined the effects of the breakage on the subsequent growth of the basal rhizome that remained connected to the parent clone after severing the apical portion to determine whether any reduction in number of ramets in the apical portion due to severing was compensated by increased ramet production in the basal portion. To this end, three rhizomes within each block were severed at three different positions relative to the apex as described above and the remaining three rhizomes were left intact. The four ramets closest to the break on the basal portion were numbered consecutively from the youngest (nearest to the breakage point) to the oldest ramet. The internode of the oldest ramet was marked with a cotton thread to facilitate its identification at the end of the experiment. Undisturbed rhizomes were marked along the rhizome at the same distances as severed ones. At the beginning of the experiment, the number of ramets and length of rhizome in the apical portions were recorded.

At the end of the two experiments (November 2010), survived rhizome portions from the first experiment were carefully excavated from the substrate to avoid root damage and transported to the laboratory. The length of rhizome, the number of new vertical shoots and buds, the number and length of roots, the length of the longest shoot, the length of vertical internodes of shoots and the number of new ramets produced by each apical portion in disturbed rhizomes or by an equivalent part in undisturbed rhizomes were recorded. The increase in rhizome length relative to initial length was also calculated and expressed as percentage. Plants were then separated into shoots, roots and rhizomes, and all plant parts dried at 70 °C and weighted. Total dry mass was calculated as the sum of all parts dry masses. To examine plant response in terms of resource allocation, the root to shoot ratio was calculated by dividing root by shoot biomass (g g^-1^ DW). Because disturbance can affect tissue density without an apparent modification of growth rate, we also determined the specific length of shoots, rhizome and roots (m g^-1^ DW), which are considered indicators of the dry mass costs of tissue production [8] of each apical portion. For the second experiment, the total number of newly produced branches and their position on nodes relative to the breakage point, and the number of new ramets in each basal portion of disturbed rhizomes or by an equivalent part in intact rhizomes were recorded non-destructively in the field. The final length of the rhizome and the number of newly produced ramets in the apical portion of disturbed and undisturbed rhizomes were also determined. Finally, the angle formed by each branch produced on basal portions was measured to determine whether the direction of growth of newly produced rhizome axes deviated from that of the original rhizome after severing apical rhizome portions, and therefore re resulted in alterations of the distribution of ramets in space.

### Statistical analysis

Disconnected apical portions that contained only two ramets at the start the experiment were damaged or desiccated so were omitted from analyses. To evaluate whether apical portions assigned to the different treatment combinations were similar in morphology, data recorded at the start of the experiment for each of the two categories of survived apical portions (intermediate and long) were separately analyzed according to a randomized block design that included the orthogonal factors block (three levels, random) and treatment combination (four levels, increased burial and rhizome severed, ambient conditions and rhizome severed, increased burial and rhizome let intact, ambient conditions and rhizome left intact, random) throughout multivariate analysis of variance by permutation, PERMANOVA [43]. Separate Fisher’s exact tests were performed for each of the three categories of apical portions to test for differences in survival between severed and unsevered rhizomes at the end of the experiment. Final data on morphological (number of new shoots, buds and roots, length of the longest shoot and length of vertical internodes) and growth-related characters (biomass of shoots, rhizomes and roots) were analyzed through PERMANOVA according to a mixed model design that included the orthogonal factor rhizome status (two levels, fixed; disturbed and undisturbed), breakage localization (two levels, fixed; sixth and twelfth internode), burial regime (two levels, fixed; ambient and increased frequency and intensity) and block (three levels, random). Since significant effects were detected in PERMANOVA on final data, separate ANOVAs were performed for the investigated variables according to the same model. Separate ANOVAs were also conducted on total plant biomass, relative increase in rhizome length, root to shoot ratio and specific length of shoots, rhizome and roots. In the second experiment, for each breakage position treatment the total number of new ramets produced by basal and apical portions combined in disturbed rhizomes was calculated and compared to that produced in equivalent portions in intact rhizomes by pair one-tailed *t*-test [44]. As no effect of block was detected for any of the variables examined in multivariate and univariate analyses, block was dropped as a factor from analyses.

Prior to PERMANOVA, data were normalized and dissimilarities calculated as Euclidean distances. Significance levels were calculated from 9999 permutations of the residuals under the reduced model. Whenever possible, *post hoc* pooling of mixed terms of the model was performed to increase analysis power [44]. When a significant effect was found, *post hoc* pair-wise comparisons (PERMANOVA *t* statistic and 999 permutations) were used to distinguish between means. For some terms, there were not enough permutable units to get a reasonable test by permutation, so *P*-values were obtained using a Monte Carlo random sample from the asymptotic permutation distribution [43]. Statistically significant terms were checked for differences in multivariate group dispersion with the permutational analysis of multivariate dispersions (PERMDISP); pair-wise comparisons of multivariate dispersion were also performed between all couples of groups. Prior to performing ANOVAs, data were tested for normality and homogeneity of variances, and transformed if necessary. Whenever data transformation failed to achieve homogeneity of variances, the analysis was performed on untransformed data with α = 0.01 [45]. When significant effects were detected, means were compared through the Student-Newman-Keuls (SNK) test [45]. As for the multivariate analysis, *post hoc* pooling of mixed interaction term was applied whenever possible. PERMANOVA and PERMDISP were run through PRIMER v6 (Primer-E Ltd., Plymouth [45]) with PERMANOVA add-on software, while statistical software R version 2.12.2 [46] and R package “GAD” [47,48] were used for ANOVAs.

## Results

### Characteristics of naturally detached rhizomes

**Figure 3:**
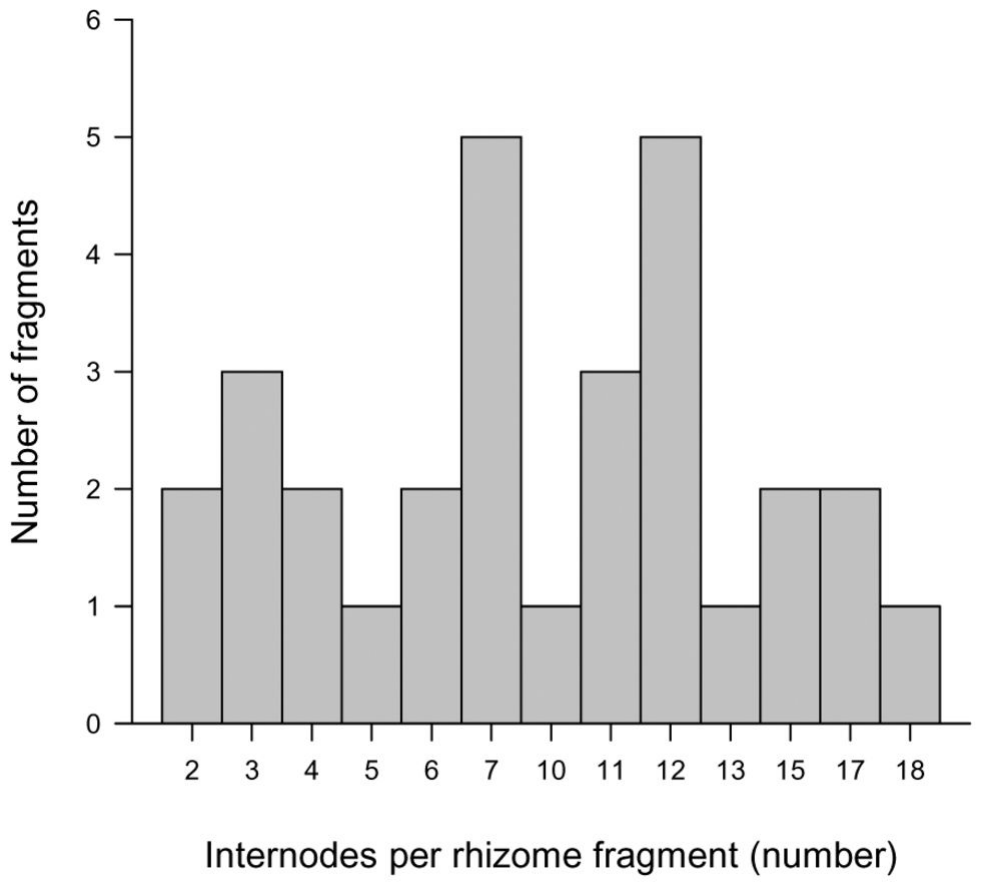
Number of internodes per naturally detached rhizome fragment of *Sporobolus* *virginicus* . Number of internodes per horizontal rhizome fragment naturally detached by storms at the study system.

The naturally detached rhizome fragments collected along the dune system varied greatly in length from 14 to 61 cm with an average of 38.6 ± 4.2 (mean ± SE) cm. The number of internodes per fragment ranged from 2 to 18 (mean, 9 ± 0.5). In most fragments (88%), the breakage had occurred between the second and twelfth internode proximal to the apex (Figure 3).

### Impact of breakage, breakage position and burial regime on the apical portion

The effect of breakage on survival was marginally significant (*P* = 0.05) for apical portions that included two interconnected ramets. None of these portions survived to the end of the experiment when disconnected from parent clones whereas all portions left connected to the parent clone survived and had produced new ramets (Table 1). All intermediate and long apical portions both disconnected and connected to clones survived to the end of the experiment. No new primary ramet was produced on intermediate or long disconnected apical portions, while there were four to nine new ramets in equivalent parts of intact undisturbed rhizomes (Table 1). Overall, both intermediate and long disconnected apical portions significantly differed from equivalent portions in intact rhizomes, and there was a significant difference among intermediate and long portions but only in intact rhizomes (significant interaction between breakage localization and rhizome status (Table 2)). Apical portions were also significantly affected by burial as main factor (Table 2). Since no differences in multivariate dispersion were detected among significantly differing groups (PERMDISP test for burial regime: *F*_1,22_ = 0.03, *P* = 0.881; PERMDISP test for internode position x rhizome status term: *F*_3,20_ = 3.621, *P* = 0.102), the observed effects were ascribed to the investigated factors rather than to heterogeneity in multivariate dispersion.

**Table 1:** Rhizome length of apical portions at the start of the first experiment, and rhizome length and number of new ramets produced by the apical meristem of apical portions of *Sporobolus* *virginicus* horizontal rhizomes subjected to different treatment combinations at the end of the experiment.

		**Beginning of the experiment**	**End of the experiment**	
**Rhizome status**	**Internode**	**Rhizome length (cm)**	**Rhizome length (cm)**	**No. new ramets**
Undisturbed, no buried	3th	17.6 ± 0.6	34.1 ± 13.5	3 ± 1
Undisturbed, no buried	6th	26 ± 4.5	34 ± 11.8	5.3 ±1.2
Undisturbed, no buried	12th	72.6 ± 9.6	91.8 ± 11.6	6.6 ± 1.4
Undisturbed, buried	3th	18.7 ± 1.1	42.6 ± 5.8	4 ± 0.5
Undisturbed, buried	6th	31.2 ± 8.8	54.2 ± 13.9	8 ± 0.5
Undisturbed, buried	12th	52.2 ± 3.9	84.9 ± 5.8	7 ± 1
Disturbed, no buried	3th	20.4 ± 4	Nd.	Nd.
Disturbed, no buried	6th	18.2 ± 5.1	26.9 ± 5.2	0
Disturbed, no buried	12th	36.6 ± 2.6	47.7 ± 1.2	0
Disturbed, buried	3th	18.7 ± 4.2	Nd.	Nd.
Disturbed, buried	6th	20.3 ± 3.7	25.1 ± 4.6	0
Disturbed, buried	12th	55.4 ± 3.8	60.7 ± 2	0

Nd: not determined because these parts died during the experiment. Data from the three blocks are pooled. *n* = 3

**Figure 4:**
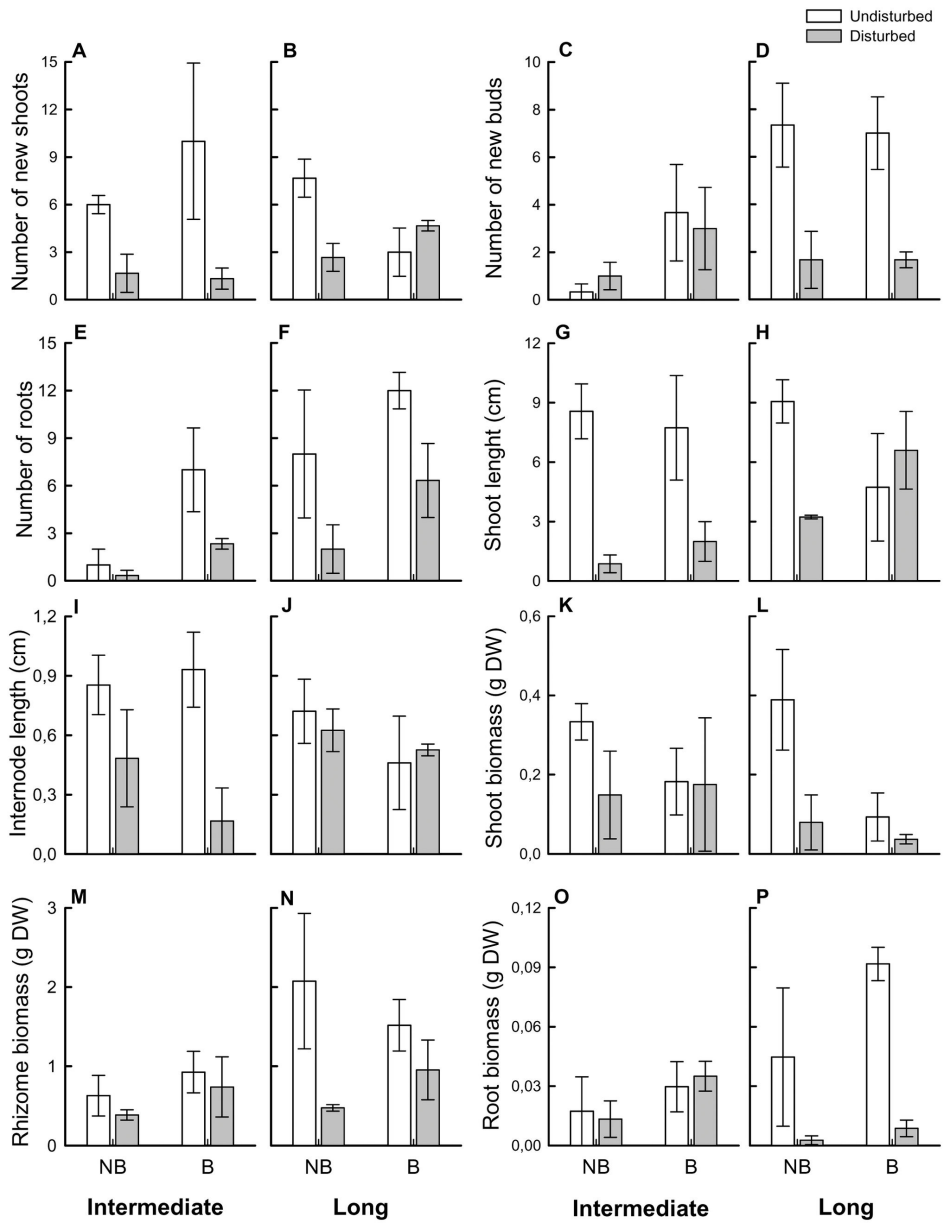
Morphological variables of apical rhizome portions of *Sporobolus* *virginicus* subjected to different treatments. Mean (± ES) number of new shoots (A, B), number of new buds (C, D), number of roots (E, F) length of the longest shoot (G, H), vertical internode length (I, J), and biomass of shoots (K, L), rhizomes (M, N) and roots (O, P) of apical rhizome portions experimentally severed at the sixth internode (intermediate portions) or twelfth internode (long portions) from the apex and equivalent parts in undisturbed horizontal rhizomes subjected to ambient or increased burial conditions, at the end of the experiment (*n* = 3). NB: no burial, ambient conditions. B: increased burial frequency and intensity. Data from the three blocks are pooled. n = 3.

**Figure 5:**
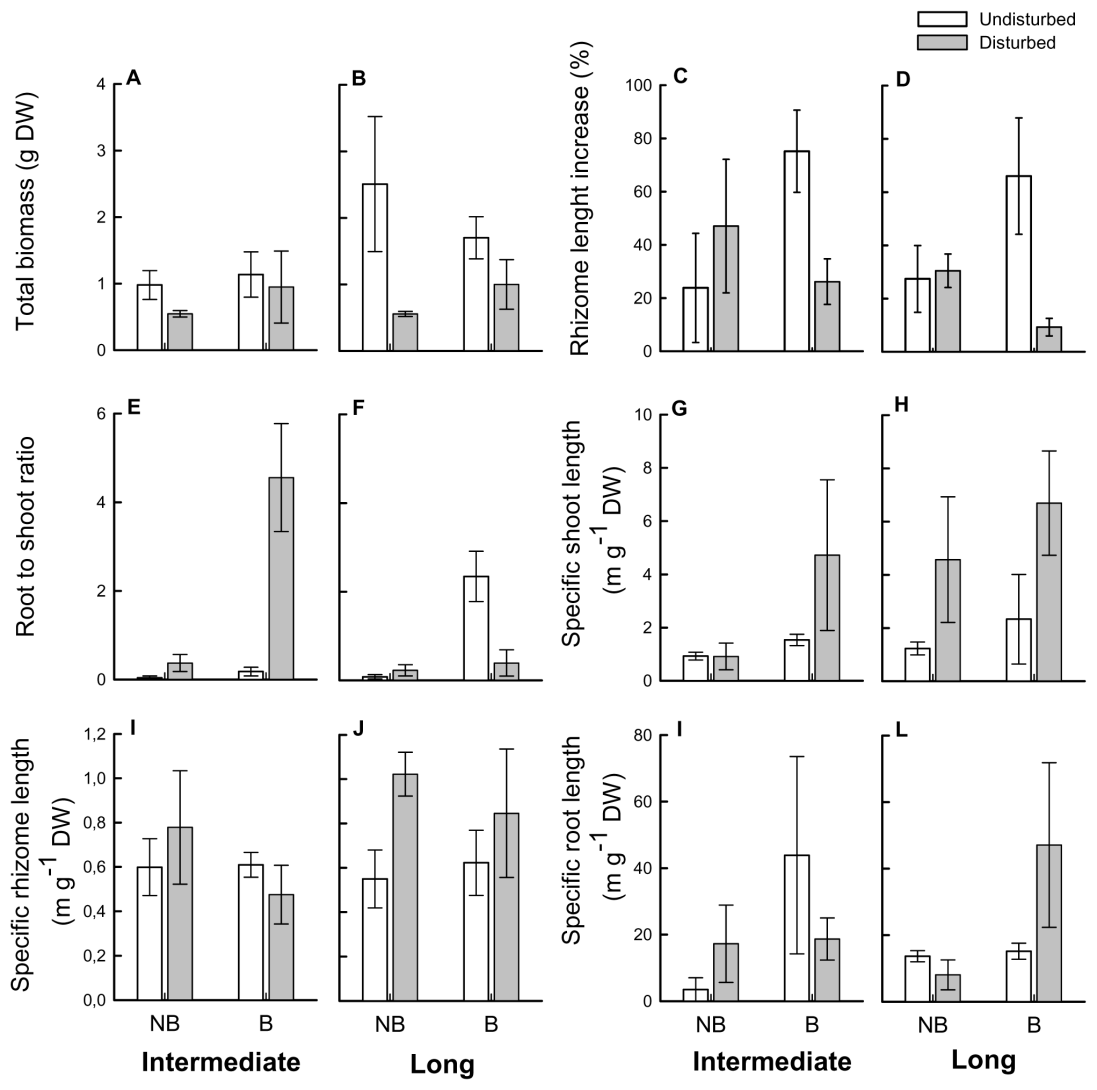
Growth-related variables of apical rhizome portions of *Sporobolus* *virginicus* subjected to different treatments. Mean (± ES) total biomass (A, B), relative rhizome length increase (C, D), root to shoot ratio (E, F), specific shoot length (G, H), specific rhizome length (I, J) and specific root length (K, L) of apical rhizome portions experimentally severed at the sixth internode (intermediate portions) or twelfth internode (long portions) from the apex and equivalent parts in undisturbed horizontal rhizomes subjected to ambient or increased burial conditions, at the end of the experiment (*n* = 3). NB: no burial, ambient conditions. B: increased burial frequency and intensity. Data from the three blocks are pooled. n = 3.

ANOVAs on individual morphological and growth-related characters showed that in disconnected apical parts the number of roots (Figure 4E,F), the biomass of shoots (Figure 4A,B) and the total biomass (Figure 5A,B) were significantly reduced while the specific shoot length (Figure 5G,H) was increased compared with those of equivalent parts in intact rhizomes, regardless of breakage position and burial regime (Table 3). A decrease in the number of new shoots (Figure 4A,B), length of shoots (Figure 4G,H) and vertical internodes (Figure 4I,J) and an increase in root to shoot ratio (Figure 5E,F) were detected in disconnected intermediate apical portions, while a reduction in the biomass of roots (Figure 4E,F) and number of new buds (Figure 4C,D) was recorded in long apical portions (significant interactions between rhizome status and breakage localization; Table 3). Increased burial enhanced the number (Figure 4E,F) and biomass of roots (Figure 4O,P), specific root length (Figure 5K,L) and root to shoot ratio (Figure 5E,F) of apical portions regardless of rhizome status (Tables 3 and 4), indicating a greater biomass allocation to root tissues. Burial also induced greater rhizome length increase relative to initial length (Figure 5C,D) in apical portions but only in intact rhizomes (significant interaction between rhizome status and burial regime, Table 4). Shoots were longer in undisturbed rhizomes compared to disturbed ones (Figure 4G,H) only under ambient conditions (significant interaction between rhizome status and burial regime, Table 3). No significant effect of any of the investigated factors was detected for specific rhizome length (Table 3, Figure 4I,J).

**Table 2:** Multivariate analysis (PERMANOVA) of the number of new shoots, buds and roots, length of the longest shoot, length of vertical internode, and biomass of shoots, rhizome and roots of *Sporobolus* *virginicus* apical rhizome portions at the end of the experiment.

**Source of variation**	**d.f.**	**MS**	**Pseudo-*F***	***P***
Internode position = I	1	12.95	2.48	0.025
Rhizome status = R	1	38.48	1.87	0.284
Burial = B	1	11.91	2.28	0.038
I x R	1	20.60	3.94	0.001
I x B	1	3.11^a^		
R x B	1	5.91	1.13	0.352
I x R x B	1	6.18^a^		

^a^ Denotes post-hoc pooling, *P* > 0.25; new *F*-values are given for those tested against the pooled term. Each test was based on 9999 permutations of residuals under the reduced model.

**Table 3:** Analysis of variance (ANOVA) of the number of new shoots, buds and roots, length of the longest shoot, vertical internode length, and biomass of shoots, rhizome and roots of *Sporobolus* *virginicus* apical rhizome portions at the end of the experiment, and results of SNK tests.

		**Number of new shoots**		**Number of new buds**		**Number of roots**	
**Source of variation**	**d.f.**	**MS**	***F***	**MS**	***F***	**MS**	***F***
Internode position = I	1	0.09	0.15	35.04	6.74*	117.04	9.92**
Rhizome status = R	1	5.19	1.90	45.38	1.00	108.38	9.19**
Burial = B	1	0.02	0.04	9.38	0.78	100.04	8.48**
I x R	1	2.73	4.55*	45.38	8.73*	15.04^a^	
I x B	1	0.45^a^		12.04	2.32	0.04^a^	
R x B	1	0.60	0.29	0.38	0.07	5.04	0.43
I x R x B	1	2.06	3.44	1.04^a^		7.04^a^	
Residual	16	0.61^a^		5.46^a^		12.63^a^	
SNK test		6th:D>U		12th:U>D, U:12th > 6th		12th > 6th, U>D, B>NB	
Transformation		None		None		None	
		**Shoot length**		**Vertical internode length**		**Shoot biomass**	
**Source of variation**	**d.f.**	**MS**	***F***	**MS**	***F***	**MS**	***F***
Internode position = I	1	0.50	1.45	0.004	0.05	0.02	0.87
Rhizome status = R	1	4.78	2.31	0.51	1.11	0.12	4.63*
Burial = B	1	0.01	0.03	0.13	1.55	0.08	3.19
I x R	1	2.07	5.94*	0.46	5.26*	0.01^a^	
I x B	1	0.07^a^		0.01^a^		0.02^a^	
R x B	1	1.67	4.79*	0.02	0.23	0.07	2.77
I x R x B	1	0.45^a^		0.12^a^		0.002^a^	
Residual	16	0.36^a^		0.09^a^		0.03^a^	
SNK test		6th:U>D, NB:U>D		6th:U>D		U>D	
Transformation		Sqrt(x+1)		None		None	
		**Rhizome biomass**		**Root biomass**			
**Source of variation**	**d.f.**	**MS**	***F***	**MS**	***F***		
Internode position = I	1	0.41	5.60*	0.17	0.10		
Rhizome status = R	1	0.54	3.22	4.52	0.39		
Burial = B	1	0.08	1.07	13.85	8.68**		
I x R	1	0.17	2.28	11.52	7.22*		
I x B	1	0.02^a^		0.19^a^			
R x B	1	0.06	0.84	0.09	0.06		
I x R x B	1	0.05^a^		0.01^a^			
Residual	16	0.07^a^		1.78^a^			
SNK test		12th > 6th		B>NB, 12th:U>D			
Transformation		None		None			

^a^ Denotes post-hoc pooling, *P* > 0.25; new *F*-values are given for those tested against the pooled term. D: disturbed rhizomes. U: undisturbed rhizomes. NB: no burial, ambient conditions. B: increased burial frequency and intensity. * *P* < 0.05; ** *P* < 0.01.

**Table 4:** Analysis of variance (ANOVA) of total biomass, relative rhizome length increase (%), root to shoot ratio, specific shoot length, specific rhizome length and specific root length of *Sporobolus virginicus* apical rhizome portions at the end of the experiment, and results of SNK tests.

		**Total biomass**		**Rhizome length increase**		**Root to shoot ratio**	
**Source of variation**	**d.f**	**MS**	***F***	**MS**	***F***	**MS**	***F***
Internode position = I	1	0.94	3.20	0.01	0.01	0.02	0.02
Rhizome status= R	1	3.20	10.86**	1.26	1.22	1.20	0.19
Burial = B	1	0.09	0.31	1.06	0.46	8.85	7.47*
I x R	1	0.42^a^		0.89^a^		6.22	5.25*
I x B	1	0.0^a^		2.28	2.20	0.03^a^	
R x B	1	0.28	0.96	7.28	6.99*	0.42	0.12
I x R x B	1	0.12^a^		0.001^a^		3.37	2.84
Residual	16	5.04^a^		1.11^a^		1.26^a^	
SNK test		U>D		U:B>NB, B:U>D		6th:D>U, B>NB	
Transformation		Log (x)		Log (x)		None	
		**Specific shoot length**		**Specific rhizome length**		**Specific root length**	
**Source of variation**	**d.f**	**MS**	***F***	**MS**	***F***	**MS**	***F***
Internode position = I	1	17.01	2.41	0.13	1.58	12.77	1.51
Rhizome status= R	1	44.34	6.29*	0.21	1.30	0.36	0.04
Burial = B	1	21.92	3.11	0.06	0.74	38.98	4.62*
I x R	1	7.68^a^		0.16	2.01	5.03^a^	
I x B	1	0.53^a^		0.01^a^		11.67^a^	
R x B	1	6.70	0.95	0.12	1.50	0.02	0.00
I x R x B	1	1.76^a^		0.002^a^		15.41^a^	
Residual	16	7.75^a^		0.09^a^		8.02^a^	
SNK test		U>D				B>NB	
Transformation		None		None		None	

^a^ Denotes post-hoc pooling, *P* > 0.25; new *F*-values are given for those tested against the pooled term. U: undisturbed, intact rhizomes. D: disturbed, severed rhizomes. NB: no burial, ambient conditions. B: increased burial frequency and intensity. * *P* < 0.05; ** *P* < 0.01.

### Impact of breakage on basal and apical portions combined

Severing the rhizome proximal to the third internode had no stimulatory effect on nodes on the basal rhizome portion remained connected to clone. Instead, severing the rhizome at the sixth or twelfth internode promoted the emergence of a new lateral branch in respectively 33.3% and 66.6% of the basal portions (Table 5). Newly produced branches emerged from the second or third node closest to the breaks. No branch emerged from the first node. Nodes at equivalent positions on the basal portion of intact rhizomes remained inactive (Table 5). New branches formed an angle with the main rhizome axis of 46.6 (± 22.8; *n* = 3). At the end of the experiment, the length of branches produced after severing proximal to the sixth internode ranged from 0.9 and 2.4 cm and that of branches formed after severing at the twelfth internode was 3.1 cm (Table 5). In disturbed rhizomes, the total number of new ramets produced by the basal and apical portions in combination was always significantly lower than that produced by equivalent segments in intact rhizomes (Table 5) over exactly the same period (*t* = -3.14, *P* = 0.03 for rhizome severed at the third internode, *t* = -3.47, *P* = 0.02 for rhizome severed at the sixth internode, and *t* = 8.84, *P* = 0.001 for rhizomes severed at the twelfth internode).

**Table 5:** Mean (± SE) rhizome length of apical portions, number and length of new branches and new ramets in basal portions and total number of new ramets on apical and basal portions combined for *Sporobolus virginicus* horizontal rhizomes subjected to different treatment combinations the end of the second experiment.

		**Apical portion**	**Basal portion**			**Basal plus apical portion**
**Rhizome status**	**Internode**	**Rhizome length (cm)**	**No. branches**	**Branch length (cm)**	**No. new ramets**	**Total ramet number**
Undisturbed	3th	34.2 ± 14.1	0		0	10.3 ± 2.3
Undistrurbed	6th	34.6 ± 10	0		0	11.3 ± 1.2
Undistrurbed	12th	94.3 ± 7	0		0	18.6 ± 0.3
Disturbed	3th	Nd.	0		0	0
Disturbed	6th	25.5 ± 2	0.6 ± 0.3	1.1 ± 0.7	0.6 ± 0.6	6.6 ± 0.5
Disturbed	12th	49.9 ± 6.1	0.3 ± 0.3	3.1	0.6 ± 0.6	12.6 ± 0.6

Data from the three blocks are pooled. *n* = 3.

Nd: not determined because these parts died during the experiment. Data from the three blocks are pooled. *n* = 3

## Discussion

Recently, a number of studies have investigated the importance of physiological integration of rhizomatous plants to persistence in stressful habitats such as mobile dunes [7–10,12–14,22,49]. Similarly to the present experiment, these studies included the interactive effects of fragment size and sand burial or water submergence, or try to detect the role of apical dominance after fragmentation processes. However, this is the first study that addressed the consequences of breakage of rhizome connections on the whole growth and spatial colonization pattern of damaged clones under increased burial conditions in the field, and therefore under a more realistic scenario.

The results of the first experiment demonstrate that rhizome breakage, and therefore the loss of clonal integration, affected the survival and subsequent growth of apical portions, especially smaller ones. Indeed, apical portions severed proximal to the third internode did not survive. These portions contained young, unrooted ramets that were not capable of taking up water and nutrients, and an internal support through physiological integration is thus essential for their survival and growth. Instead, apical portions severed at sixth (intermediate portions) or twelfth internode (long portions) persisted, but they were unable to produce new ramets and exhibited decreased total biomass and thinner shoots. In addition, intermediate portions showed decreased shoot production, shoot length and increased root to shoot ratio and long portions exhibited reduced production of new buds and root biomass. These findings are consistent with the results of previous studies on the effects of burial, stolon internode length and their interaction on survival and growth of an invasive clonal herb, *Alternatheraphiloxeroides* [12–14], and suggest that longer apical portions might survive after disconnection from parent clone owing to the remobilization and reuse of reserves stored in rhizome internodes. Previous studies indicted that that internodes of clonal plants contain storage materials that can be remobilized by ramets when necessary [9,11–13]. They also indicates that both the activity of the apical meristem and the growth of ramets of *S* *. virginicus* even situated at large distances from the rhizome apex (up to 70 cm) depend strongly on the resources translocated from the parent clone. On the other hand, the lack of significant effects on the relative increase in length of apical portions following separation from the parental rhizome suggests that rhizome elongation was maintained near to an optimal rate through extension of pre-existing internodes. The resources needed to support this growth were presumably achieved through a reduction in the production costs of shoots since more shoot length were produced with the same amount of biomass [5]. Hence, the performance of apical rhizomes was reduced by disturbance. Only detached apical fragments of larger size seemed to have the potential to establish new clonal patches under ambient conditions. Further longer studies are needed to test if the recruitment of new ramets is only temporary inhibited by rhizome severing and restart in the subsequent growing season.

Under increased burial, both disconnected and connected apical portions allocated more biomass to roots independently on breakage position. The production of new roots under burial conditions has been previously observed in some dune plants, and it could be attributed to increasing nutrients (or moisture) content decreasing plant aeration status due to sand deposition [3]. Surprisingly, no consistent burial effect on shoot length or vertical internode length was detected here. This is in contrast to that previously observed in young clones of *S* *. virginicus* growing in the same study area after exposure to a similar burial regime [41]. As burial reduces net photosynthesis and carbohydrate production in plants, elongation of vertical structures and regeneration of the lost photosynthetic capacity is an essential mechanism for surviving in dune habitats [3,6,26]. Indeed, the most common morphological response observed in dune plants to burial is a shift in the allocation of resources from below-to above ground plant parts as predicted by the classic theory of biomass allocation[3,6,8,25,26]. . Instead, in this study we found a greater rhizome elongation in survived apical portions connected to the parent clone (20-30% vs. 70-80% respect to the original size) and no significant increase on disconnected ones. The increase in the biomass allocated to roots in the connected ramets could be in accordance with the division of labour theory in clonal plants [50]. Rhizome elongation in response to burial has been previously documented only for another rhizomatous dune species, *Ammophila* *breviligulata* L. Link [18]. Spreading biomass laterally rather than vertically could be an important alternative adaptive strategy evolved in clonal plants to escape from unfavourable zones [15–17,21] such as mobile dune habitats and reduce the risk of death to the whole plant from burial. This mechanism appeared, however, to be insufficient to support higher rhizome elongation rates required to escape from increased burial once an apical rhizome portion is disconnected from the parent clone by disturbance because of the limited availability of internal pool of reserves. This supports our first hypothesis that breakage interacted, and counteracted the stimulatory effect of burial on rhizome growth. Because of this interactive effect and the incapacity to elongate vertically, survived apical rhizome fragments may be more vulnerable to any post-disturbance changes in the local environment, and thus may have no or little chance to establish new patches on active dune areas.

Results of the second experiment indicate that clones of *S* *. viriginicus* responded to rhizome breakage and removal of apical portions by enhancing branching rates on the portions that remained connected to the parent clone. Indeed, the loss of the main rhizome apex after severing apical portions that included five or 11 interconnected ramets was matched by the outgrowth of a lateral branch from nodes distant from breaks on the basal portions remained connected to clone. This sprouting response confirms our second hypothesis and suggests that the inhibitory effect of the apical meristem on lateral meristems in the study species is effective at large distances (up to 70 cm from the apex). The lack of response to the removal of apical rhizome portions containing only two ramets at the time of severing was probably due to the physiological immaturity of ramets closest to the break on the basal portions or the low supply of resources from nodal roots which have been found to play a role in influencing axillary bud outgrowth in plagiotropic clonal plants [51]. The reduction in number of ramets due to the removal of an apical portion, however, was never compensated by the production of new ramets at least in the current growing year. Moreover, the replacement of the original apex by a new branch caused a change in the mean direction of about 46°C respect to the original growth trajectory of rhizomes. *S* *. virginicus* clones can therefore regenerate after removal of apical rhizome segments, but the this kind of this disturbance may cause a significant net reduction in mean number of ramets, from 29 to 100% of the ramet number that would be produced by an intact rhizome under ambient conditions. This effect was estimated on the basis of a single damage event imposed on an individual rhizome, but in nature a disturbance event may affect much of the rhizomes of a clone. Recurrent disturbance events may clearly have a greater impact, leading to increased loss and thus reduced rates at which clones grow outwards along with altered spatial positioning of ramets with possible circled backs of rhizomes due to deviation from the original growth trajectory.

In conclusion, the study provides novel evidence that the breakage of horizontal rhizomes by disturbance and its interaction with increased burial can affect vegetative regeneration, spread capacity and clonal architecture of dune plants. In particular, suppression of clonal recruitment along with decreased total biomass and rhizome elongation in the apical portion of *S* *. virginicus* following separation from the parent clone may reduce the chance of successful establishment of new patches under increased burial conditions. Concomitantly, the overall loss of ramets and the change of growth direction of the rhizome that remained connected to the parent clone following the removal of an apical portion may reduce the potential zone of ground exploration of the damaged clone, preventing colonization of active dunes. As *S* *. virginicus* is a pioneer species that plays an important role in stabilizing sand, reduced horizontal spread from disturbances could have implications for the stability of mobile dunes. The risk of detrimental interactive effects of increased burial and abiotic changes (such as nutrient availability) on dune plants in the future has been previously showed [41]. The evidence here of non-independent effect of burial and natural disturbance further highlights the importance of exploring greater in detail interactions between physically-driven changes in dune plants to make advancements in predicting how global change will affect the structure of plant communities and stability of dunes in future [52–54]. Human activities such as trampling by foot and vehicles and beach-cleaning operations that cause rhizome fragmentation and the artificial structures that modify sand transport may also affect dune plants [55,56]. Therefore, in future more attention should be paid to control human frequentation and activities in mobile dune areas in order to minimize the risk of synergic deleterious effects among anthropogenic disturbances and environmental alterations associated with global change on plant populations.
